# The seemingly innocuous presentation of metastatic pancreatic tail cancer: a case report

**DOI:** 10.1186/s13256-019-2125-5

**Published:** 2019-06-11

**Authors:** Milton Rahman, Letora Washington

**Affiliations:** 1 Touro University California, Vallejo, CA USA; 2 St. John’s Episcopal Hospital, Far Rockaway, NY USA

**Keywords:** Pancreatic tail cancer, Pancreatic tail adenocarcinoma, Pancreatic cancers of the tail, Pancreatic adenocarcinoma of the tail

## Abstract

**Background:**

Pancreatic cancers of the tail have an especially poor prognosis due to their late detection. An earlier diagnosis depends on a better understanding of the clinical course of the disease; however, much of the current literature focuses on pancreatic head adenocarcinomas owing to their higher incidence. Thus, we add our case report to the current literature of pancreatic tail cancers in the hope of aiding earlier detection. We present an interesting case of a patient who initially presented with innocuous abdominal pain and a single episode of vomiting who was subsequently diagnosed with metastatic pancreatic tail cancer.

**Case presentation:**

A 56-year-old Hispanic man with a past medical history of alcohol and cocaine abuse was initially evaluated in our clinic after presenting to the emergency department with sudden onset of abdominal pain and one episode of emesis. On further questioning, he stated that he had been experiencing dull, intermittent left back pain for the past 2–3 years. Laboratory tests were performed, which showed that the patient had new-onset diabetes, and imaging revealed a pancreatic tail mass with metastases to the liver. Biopsy confirmed the diagnosis of stage IV metastatic pancreatic tail adenocarcinoma. During follow-up 1 month later, the patient reported that he had been largely asymptomatic since his hospital admission; however, his left back pain had increased in severity. He was then started on a FOLFIRINOX chemotherapy regimen (5-fluorouracil/leucovorin, irinotecan, and oxaliplatin).

**Conclusions:**

There are many pitfalls in the diagnosis of pancreatic cancer, especially pancreatic tail cancer due to its vague symptoms. Thus, pancreatic cancer of the tail often presents late with a very poor prognosis. Because there is currently no widespread screening for pancreatic cancer, it is often difficult for practitioners to identify pancreatic tail cancers. Current research suggests that there is a strong association between new-onset diabetes after the age of 50 and pancreatic cancer, and tumors detected at the onset of diabetes are favorable to resection. Pancreatic cancer has also been shown to be associated with certain risk factors, such as smoking, high body mass index, chronic pancreatitis, and a family history of pancreatic cancer. Thus, when patients with presentations similar to our patient’s with new-onset diabetes after the age of 50, along with vague symptoms such as back or abdominal pain as well as the presence of risk factors, we suggest that it is beneficial for practitioners to maintain a high index of suspicion for pancreatic cancer.

## Introduction

Pancreatic cancer is a cancer with a low incidence but a high mortality rate. It accounts for only 3% of new cancer cases each year; however, it is the fourth leading cause of cancer mortality and has a devastating 98% mortality rate [1, 2]. Pancreatic cancer is sometimes referred to as the “silent” disease because it is often asymptomatic at early stages. When symptoms do occur, they are often vague, and they vary depending on the location of the tumor [3].

Approximately 75% of all pancreatic carcinomas arise in the head of the pancreas; 15–20% arise in the body; and 5–10% arise in the tail [4]. Previous research has demonstrated meaningful differences in presentation and mortality between carcinomas arising in the pancreatic head versus in the pancreatic body and tail [5]. Tumors arising in the body and tail tend to present later, often with infiltration of adjacent organs or metastatic disease on presentation, and thus are associated with poorer survival rates than head lesions [6–8]. In the earlier stages, pancreatic tail cancer is often undiagnosed or misdiagnosed during emergency department visits due to its vague symptoms [3]. In this case report, we describe a patient with pancreatic tail adenocarcinoma who presented with mild clinical symptoms, despite imaging revealing advanced metastatic disease. It is our hope that our reporting of the symptoms as well as imaging and laboratory changes in our patient may further awareness and aid in the earlier identification of a difficult-to-diagnose cancer with a high mortality rate.

## Case presentation

A 56-year-old Hispanic man with a past medical history of alcohol and cocaine abuse was initially evaluated in our clinic after presenting to the emergency department with sudden-onset abdominal pain and one episode of emesis. The patient stated that this was the first time an episode like this had ever occurred in him, and he described the pain he had felt as epigastric in location, nonradiating, and 8/10 on a numeric rating scale. He denied any other symptoms, including weight loss, changes in appetite, and changes in stool. However, when asked about back pain, he recalled that he had been experiencing dull, intermittent left back pain for the past 2–3 years that radiated to his left rib cage at the midaxillary line. He described the pain as 4/10 in severity at its worst and denied feeling any pain on his right side.

The patient reported being a smoker in the past but that he had quit approximately 15 years earlier. He also reported using cocaine once per week and heavy drinking of liquor during certain months of the year. His past medical history was otherwise noncontributory; however, he did report inconsistent visits with his last primary care provider. His social history was significant for his occupation as a landscaper, which had caused the patient to disregard his back pain as being a work-related injury.

**Fig. 1:**
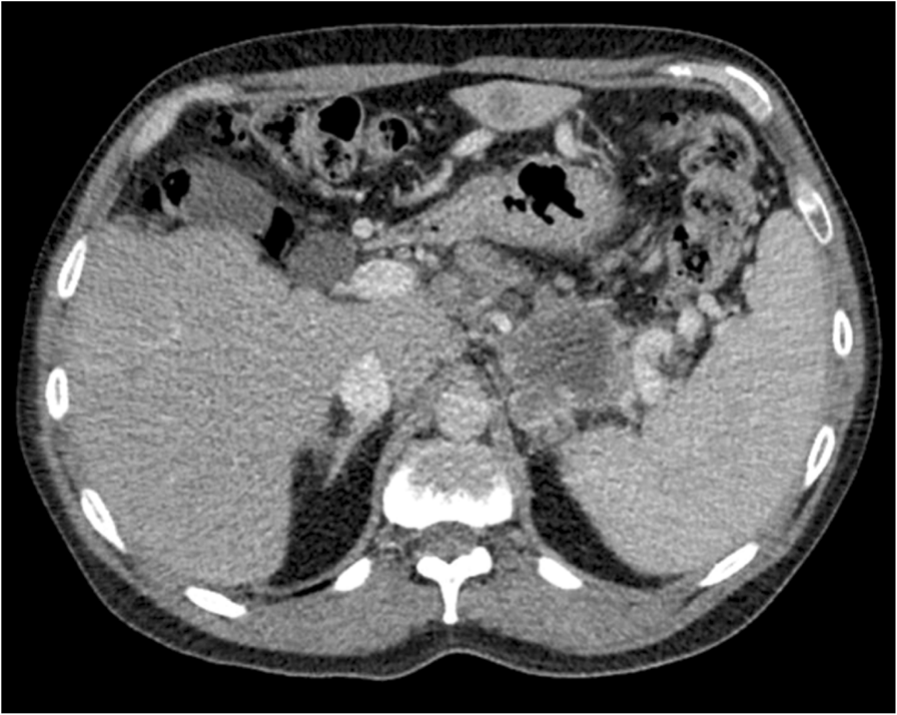
Axial plane abdominal/pelvic computed tomographic scan with intravenous contrast showing a 6.4 × 4.9-cm pancreatic tail mass inseparable from the left adrenal gland

**Fig. 2:**
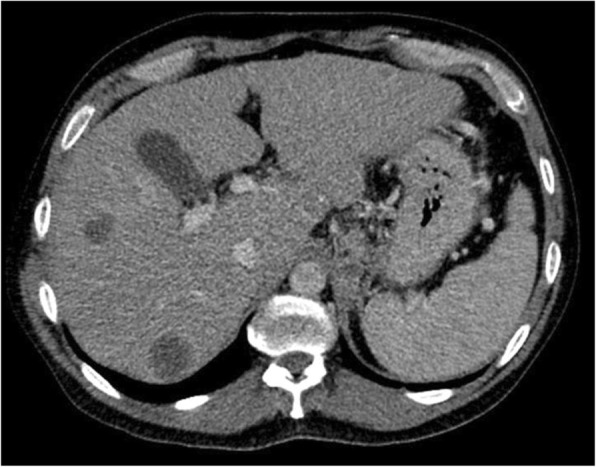
Multiple peripheral, round, enhancing hepatic lesions, with the largest measuring 3.3 × 3.1 cm

The result of his complete physical examination was unremarkable, except for his body mass index being clinically overweight at 26.6 kg/m^2^. His abdomen was soft and nondistended, and his bowel sounds were normal. Basic laboratory tests were performed during his hospital admission and revealed an elevated blood glucose level (612 mg/dl; reference range < 140 mg/dl) and an elevated hemoglobin A1C (13.3%; reference range < 5.7%). Abdominal/pelvic computed tomography (CT) with intravenous contrast revealed abnormalities suggestive of malignancy in the pancreatic tail and multiple liver metastases (Figs. 1 and 2). An elevated cancer antigen 19-9 tumor marker (15,453 U/ml; reference range < 37 U/ml) was also noted, which was strongly suggestive of pancreatic carcinoma. A CT-guided needle biopsy of the liver was performed, and results were consistent with a poorly differentiated primary pancreatic adenocarcinoma. Immunohistochemical analysis revealed neoplastic cells that were CK7^+^/CK20^−^ and positive for CDX2, MUC5AC, and PODXL1, which was highly suggestive of a primary pancreaticobiliary neoplasm. Thus, a diagnosis of stage IV pancreatic tail adenocarcinoma with multiple liver metastases was made.

During follow-up 1 month later, prior to the start of chemotherapy, the patient stated that he had been experiencing worsening left back pain; however, had not experienced any epigastric pain, nausea, or vomiting since his hospital admission. He described the pain as now constant and radiating anteriorly to the left lower ribs at the midclavicular line. The pain was rated as 8/10 on a numeric rating scale, and the patient reported that even the pressure of putting a shirt on hurt him and that he could only now lie on his right side. On physical examination, the region was exquisitely tender to palpation, but the result of the remainder of his examination was unremarkable. The patient was then started on a FOLFIRINOX chemotherapy regimen (5-fluorouracil /leucovorin, irinotecan, and oxaliplatin).

## Discussion

Pancreatic cancer is a devastating illness with a high mortality rate. Pancreatic carcinomas involving the head of the pancreas are more common and often present with symptoms such as weight loss, jaundice, dark urine, light stool color, abdominal pain, nausea, and vomiting. In contrast, pancreatic carcinomas arising from the tail are less common and present later, usually with abdominal pain, back pain, and weight loss. Pancreatic cancer of the tail is often undiagnosed or misdiagnosed and thus is associated with a higher mortality rate due to its frequent late presentation [3, 4, 7, 8]. An earlier diagnosis depends on a better understanding of the clinical course of the disease; however, much of the current literature focuses on pancreatic head adenocarcinomas due to their higher incidence. Thus, with this case report, we aim to add to the current literature of pancreatic adenocarcinomas involving the pancreatic tail to aid in their earlier detection.

Our patient with metastatic pancreatic tail adenocarcinoma initially presented with few clinical symptoms despite advanced disease. The patient experienced intermittent left back pain; however, he had disregarded it for 2–3 years until it progressively worsened. He experienced no other symptoms except for a single episode of epigastric abdominal pain and vomiting that resulted in hospital admission, at which time the patient’s disease had already advanced into stage IV metastatic disease. When the patient was seen during follow-up 1 month later, his left back pain had rapidly evolved, suggesting the rapidly infiltrating characteristics of the tumor.

There are many pitfalls in the diagnosis of pancreatic cancer, especially pancreatic tail cancer due to its vague symptoms. The presentation of our patient highlights the importance of awareness of the symptoms of pancreatic cancer and the need for further research in developing an effective screening methodology [9]. The current standards for detecting pancreatic cancer are costly and too invasive for widespread screening [1]. However, studies have demonstrated that patients diagnosed with diabetes over the age of 50 have an eightfold increased risk of having pancreatic cancer, and tumors detected at the onset of diabetes are favorable to resection [10, 11]. Pancreatic cancer has also been shown to be associated with certain risk factors such as smoking, high body mass index, chronic pancreatitis, and a family history of pancreatic cancer. This suggests that in the setting of primary care, it is beneficial for practitioners to maintain a high index of suspicion for pancreatic cancer in patients similar to ours who present with new-onset diabetes after the age of 50, especially in the setting of concurrent back or abdominal pain and the presence of risk factors. Nevertheless, more research is needed to help in earlier identification of pancreatic cancers, especially pancreatic tail adenocarcinomas.

## Data Availability

This report does not contain any data, so this section is not applicable.
